# Short-term effects of atmospheric pressure, temperature, and rainfall on notification rate of community-acquired Legionnaires' disease in four European countries

**DOI:** 10.1017/S0950268816001874

**Published:** 2016-08-30

**Authors:** J. BEAUTÉ, S. SANDIN, S. A. ULDUM, M. C. ROTA, P. BRANDSEMA, J. GIESECKE, P. SPARÉN

**Affiliations:** 1 European Centre for Disease Prevention and Control (ECDC), Solna, Sweden; 2 Department of Medical Epidemiology and Biostatistics, Karolinska Institutet, Stockholm, Sweden; 3 Statens Serum Institut (SSI), Copenhagen, Denmark; 4 Istituto Superiore di Sanità (ISS), Rome, Italy; 5 Rijksinstituut voor Volksgezondheid en Milieu (RIVM), Bilthoven, The Netherlands

**Keywords:** Environment and public health, Europe, Legionnaires' disease, surveillance

## Abstract

Legionnaires' disease (LD) is caused by the inhalation of aerosols containing *Legionella*, a Gram-negative bacteria. Previous national- or regional-level studies have suggested an impact of climate on LD incidence. The objective of this study was to investigate the effect of temperature, rainfall, and atmospheric pressure on short-term variations in LD notification rate. EU/EEA Member States report their LD surveillance data to the European Centre for Disease Prevention and Control. Community-acquired LD cases reported by Denmark, Germany, Italy, and The Netherlands with onset date in 2007–2012 were aggregated by onset week and region of residence. Weather variables were extracted from the European Climate Assessment & Dataset project. We fitted Poisson regression models to estimate the association between meteorological variables and the weekly number of community-acquired LD cases. Temperature, rainfall and atmospheric pressure were all associated with LD risk with higher risk associated with simultaneous increase in temperature and rainfall. Temperatures >20 °C were not associated with a higher risk for LD. LD cases occurring during wintertime may be associated with sources less influenced by meteorological conditions.

## INTRODUCTION

Legionnaires' disease (LD) is a severe pneumonia caused by *Legionella* spp. These Gram-negative bacteria found in freshwater environments tend to contaminate water systems [1]. The disease is not transmitted person-to-person, but people are infected by inhalation of aerosols containing *Legionella* [1]. Known risk factors for LD include increasing age, male sex, chronic lung disease, and various conditions associated with immunodeficiency [2, 3]. The incubation period of LD is thought to be 2–10 days (median 7 days) [4].

In temperate regions, LD shows a seasonal pattern with a peak during the warm season. In Europe, most LD cases are sporadic and community-acquired [5]. Previous findings have suggested an impact of climate on the number of LD cases reported [6–9]. Theoretically, any weather condition favouring the growth of *Legionella* or its presence in aerosols could potentially be associated with a higher LD incidence. There is evidence that *Legionella* can be found in almost any water environment whether natural or artificial [1]. Conversely, *Legionella* does not survive in dry environments [1]. Of note, *Legionella* are often found as intracellular parasites of certain protozoa, which may protect them in less favourable environments [10]. *Legionella* multiplies at temperatures between 25 °C and 42 °C with an optimum at 35 °C [11] but can survive without multiplying at lower temperatures [12]. Rainfall, temperature and humidity are the main factors associated with increased LD incidence that were reported in previous studies [6–9, 13–16]. The contribution of these different factors varied across studies which could be explained by different approaches or genuine differences due to local conditions. Hitherto, the largest study was carried out in The Netherlands including ~800 LD cases over 4 years [13], which showed that 4-week mean temperature, 2-week rainfall intensity, and 2-week rainfall duration were the best predictors of higher LD incidence. Another study conducted in the United States suggested that humidity and rainfall were better predictors than temperature [7]. A study performed in different countries with the same methodology could help identify common drivers for LD. The seasonal pattern of LD could be driven by a mix of higher temperatures and specific conditions in terms of humidity and rainfall that requires further investigation. In particular, Dunn *et al*. underlined the need to further explore the role of unusual conditions such as high temperatures [15]. Last, since climate change is expected to bring both an increase in heavy rainfall and higher temperatures, it is important to better understand the impact of weather on LD incidence [17].

Using a large international database, the objective of this study was to test and investigate the effect of temperature, rainfall, and atmospheric pressure on short-term variations in LD notification rate.

## METHODS

### LD data

**Fig. 1.**
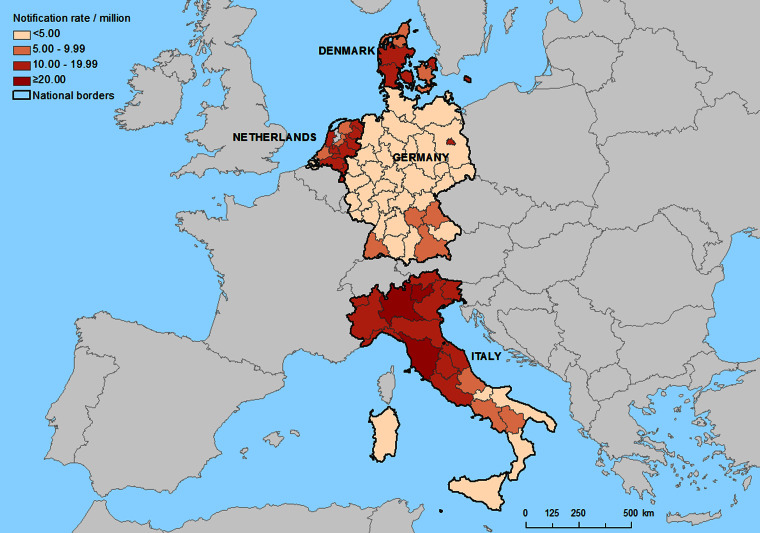
Regional average notification rates of community-acquired Legionnaires' disease cases: Denmark, Germany, Italy and The Netherlands, 2007–2012.

LD is a notifiable disease in the European Union (EU). Its surveillance is carried out by the European Legionnaires' Disease Surveillance Network (ELDSNet) under the coordination of the European Centre for Disease Prevention and Control (ECDC). ELDSNet includes all 28 EU Member States, Iceland and Norway. All LD cases meeting the EU case definition [18] are reported annually to ECDC with a set of variables including date of onset, probable setting of infection, and place of residence using the Nomenclature of Territorial Units for Statistics (NUTS). NUTS is a hierarchical system subdividing the territory of the EU into regions at three different levels [19] (Fig. 1). For the purpose of this analysis, only LD cases reported as being community-acquired were included, assuming that weather conditions at the place of residence would affect the notification rate of community-acquired LD. A community-acquired case was defined as a case with no recent travel history or admission in a healthcare facility. During the 2013 data call, four countries agreed to update their historical data with the place of residence of cases at NUTS2 level (Table 1).

**Table 1.** Study period, number of regions, population, number and annual rate of community-acquired cases of Legionnaires' disease, median and 10th–90th percentiles of weekly mean temperature, cumulative rainfall, and mean atmospheric pressure, Denmark, Germany, Italy, The Netherlands, and overall

Country	Study period	No. of regions (NUTS2)	Population (millions)	No. of LD cases	Annual rate of LD/10 million population	Median (10th-90th percentile)		
						Weekly mean temperature (°C)	Weekly cumulative rainfall (mm)	Weekly mean atmospheric pressure (hPa)
Denmark	2008–2012	5	6	286	87	9 (0–17)	10 (0–29)	1013 (1003–1023)
Germany	2007–2012	39	82	1658	34	10 (0–18)	11 (0–33)	1016 (1008–1025)
Italy	2007–2012	21	59	5148	160	13 (3–23)	9 (0–42)	1015 (1009–1022)
The Netherlands	2007–2011	12	17	869	89	11 (2–18)	12 (0–38)	1015 (1007–1025)
Overall	2007–2012	77	164	7961	85	11 (1–19)	11 (0–36)	1015 (1007–1024)

LD, Legionnaires' disease; NUTS2, Nomenclature of Territorial Units for Statistics level 2; hPa, hectopascal.

### Meteorological data

Weather variables were extracted from the European Climate Assessment & Dataset project (http://eca.knmi.nl/). Temperature (weekly average in °C), rainfall (weekly cumulative in mm), and atmospheric pressure (weekly average in hPa) were available at NUTS2 level.

### Statistical analysis

We fitted Poisson regression models to estimate the association between meteorological variables and the weekly number of community-acquired LD cases. Adjusted incidence rate ratios were obtained from the Poisson regression to estimate relative risk (RR). We controlled for seasonality, allowed for delayed effects of exposure, and ran sensitivity analyses [20]. Three meteorological exposures were explored: weekly mean temperature, cumulative rainfall, and mean atmospheric pressure. Seasonality was controlled by fitting a restricted cubic spline function of time (week number) with five knots. Spline functions are polynomial curves joined at fixed points called knots [21]. A spline function can take an arbitrarily shaped form, but requires specification of a function such as linear or cubic. Splines are thus well-suited to capture seasonality or long-term trends and therefore facilitate the detection of short-term effects. Knots locations were defined automatically following Harrell's recommended percentiles [22]. All models were further controlled for year (categorically), region (NUTS2), and an offset parameter adjusting for the population size in each region. Given the known median incubation period, a time lag of 1 week between exposure and disease onset was assumed to be the most likely. This would not prevent possible misclassification of cases with very short or long incubation periods but the assumption was deemed reasonable for most cases. Since conditions in previous weeks may also contribute to making the week of exposure more favourable for infection, we allowed delayed exposure effects up to 4 weeks before date of onset. Thus, for a given week of onset *n*, weeks *n* − 1, *n* − 2, *n* − 3 and *n* − 4 were considered for the exposure. The monthly mean temperature was also investigated. Models were compared using Akaike's Information Criterion (AIC) [23]. Models with the lowest AIC were preferred because they minimize the information loss. The meteorological variables were categorized to estimate adjusted RRs without assuming a linear association. To investigate the shape of the association between the meteorological covariates and outcome, we fitted restricted cubic spline functions with 3 d.f. for knots and the spline basis centred on the median value of the exposure. We explored interactions between categories of the three meteorological variables (mean temperature × cumulative rainfall, mean atmospheric pressure × cumulative rainfall, and mean atmospheric pressure × mean temperature) and estimated RRs in a model adjusted for year, region, and seasonality. For each meteorological variable, four categories were considered (<10 °C, 10–14 °C, 15–19 °C, and ⩾20 °C for weekly mean temperature; <10 mm, 10–19 mm, 20–29 mm, and ⩾30 mm for weekly cumulative rainfall; <1010 hPa, 1010–1014 hPa, 1015–1019 hPa, and ⩾1020 hPa for weekly mean atmospheric pressure).

Since the effect of meteorological conditions on LD notification rate may differ across the year we explored the effect of temperature and rainfall for each month separately. For temperature, we calculated the difference between the weekly mean temperature and the monthly average as observed during 2007–2012. For each month, we fitted restricted cubic spline functions with 3 d.f. for knots. Country specificities were explored by running the model for each country. We investigated unusually high temperature by using a binary variable indicating weeks with weekly average temperature ⩾20 °C (90th percentile). Finally, multiple sensitivity analyses were carried out excluding regions or countries reporting extreme values both for exposure and outcome. To address potential problems related to multicollinearity between continuous covariates we calculated the variance inflation factor (VIF) [24]. All statistical hypotheses were tested on the two-sided 5% level of significance and corresponding two-sided 95% confidence intervals (CIs) were calculated. Stata software release 14 (StataCorp. LP, USA) was used for all data management and statistical analyses.

## RESULTS

Across 77 NUTS2 regions with a total population of 164 million inhabitants, 8708 community-acquired LD cases were registered over 5–6 years, of which 8088 (93%) had available information on place of residence and date of onset. In order to include only cases with known exposure in the 4 weeks preceding the week of onset, cases with onset in the four first weeks of 2007 were excluded. Of the 7961 cases finally included in the analysis, 5148 (65%) were reported in Italy (Table 1). Within Italy, 1891 (24%) cases were located in the Lombardy region. The overall yearly notification rate was 85 LD cases/10 million population, peaking in Trento, Italy (Fig. 1).

### Rainfall

**Fig. 2.**
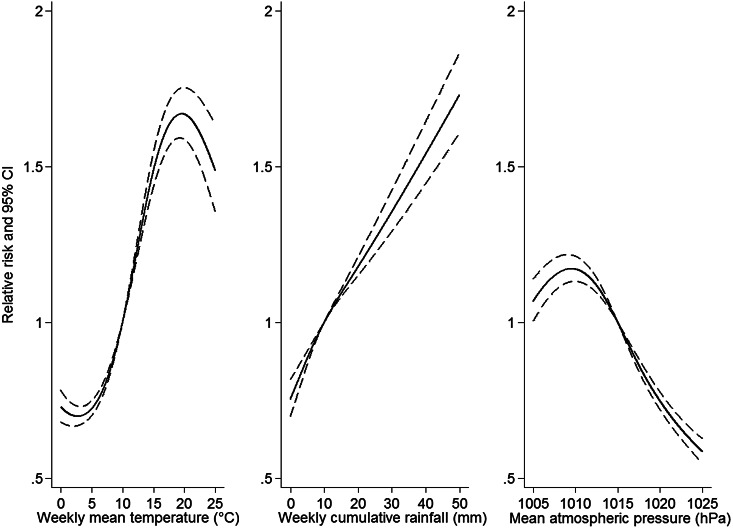
Estimated relative risk and 95% confidence intervals (CIs) of community-acquired Legionnaires' disease by weekly mean temperature (3 weeks lag) weekly cumulative rainfall (1 week lag), and weekly mean atmospheric pressure (1 week lag) in 77 regions of Denmark, Germany, Italy and The Netherlands, 2007–2012, from a model adjusted for region and population. Reference values are the median weekly mean temperature, cumulative rainfall and mean atmospheric pressure, respectively.

**Fig. 3.**
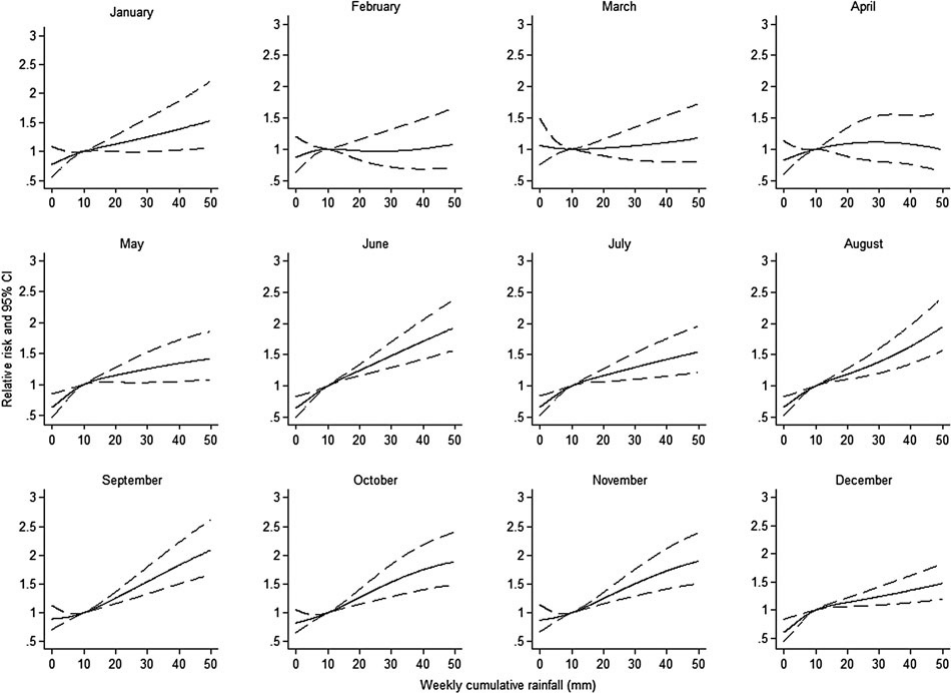
Estimated relative risk and 95% confidence intervals (CIs) of community-acquired Legionnaires' disease by month for weekly cumulative rainfall (1 week lag), in 77 regions of Denmark, Germany, Italy and The Netherlands, 2007–2012, from a model adjusted for region and population.

Weekly cumulative rainfall was positively associated with an increasing risk of LD for all lags but the association was not statistically significant for a delayed effect of 4 weeks. The association with the highest RR and lowest AIC (Table 2) was observed with a lagged effect of 1 week (RR 1·13 for every 10-mm increase, 95% CI 1·12–1·14). The flexible curve showing the relationship between weekly cumulative rainfall with 1 week lag and the RR suggested that a linear relationship existed between these two variables (Fig. 2). Flexible curves representing the overall effect of a weekly cumulative rainfall on RR by month showed different patterns across the year (Fig. 3). From February to April, the effect was limited but for all remaining months a positive association was observed.

**Table 2.** Comparison of models for cumulative rainfall, mean temperature, and mean atmospheric pressure with different delayed exposure using Akaike's Information Criterion (AIC)

Model	AIC	ΔAIC
M0: year, population, region, seasonality	25 651	
M0 + weekly cumulative rainfall no lag	25 595	−56
M0 + weekly cumulative rainfall lag 1 week	25 108	−543
M0 + weekly cumulative rainfall lag 2 weeks	25 574	−77
M0 + weekly cumulative rainfall lag 3 weeks	25 642	−9
M0 + weekly cumulative rainfall lag 4 weeks	25 650	−1
M0 + weekly mean temperature no lag	25 648	−3
M0 + weekly mean temperature lag 1 week	25 638	−13
M0 + weekly mean temperature lag 2 weeks	25 653	2
M0 + weekly mean temperature lag 3 weeks	25 629	−22
M0 + weekly mean temperature lag 4 weeks	25 643	−8
M0 + monthly mean temperature	25 651	0
M0 + weekly mean atmospheric pressure no lag	25 649	−2
M0 + weekly mean atmospheric pressure lag 1 week	25 522	−129
M0 + weekly mean atmospheric pressure lag 2 weeks	25 630	−21
M0 + weekly mean atmospheric pressure lag 3 weeks	25 653	2
M0 + weekly mean atmospheric pressure lag 4 weeks	25 649	−2

### Temperature

**Fig. 4.**
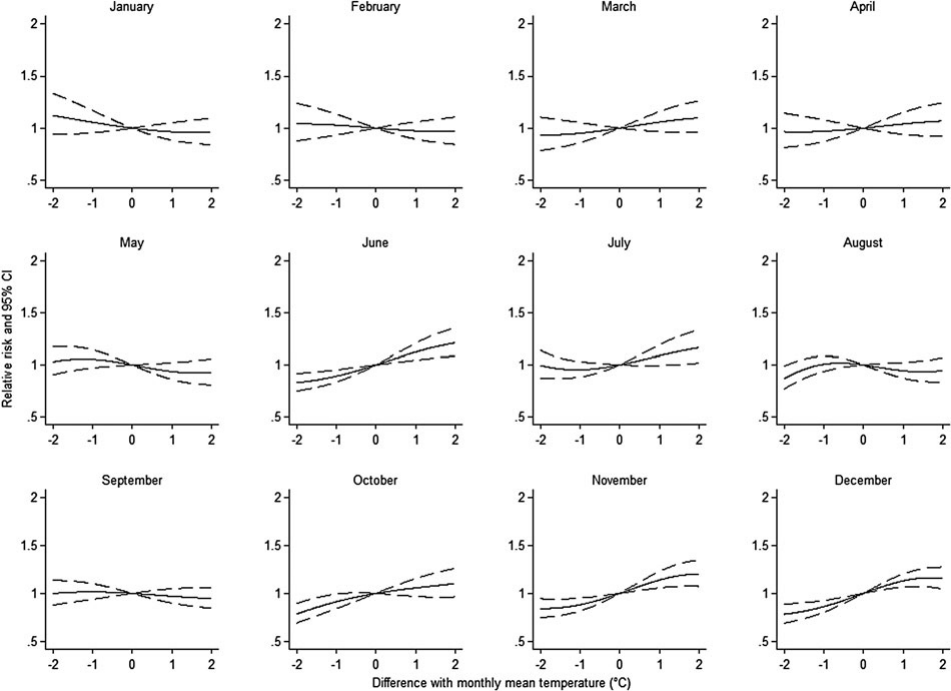
Estimated relative risk and 95% confidence intervals (CIs) of community-acquired Legionnaires' disease by month for standardized temperature (difference between 3 weeks lag mean temperature and monthly mean temperature) in 77 regions of Denmark, Germany, Italy and The Netherlands, 2007–2012, from a model adjusted for region and population.

Monthly mean temperature was positively associated with an increasing risk of LD. Weekly mean temperature was positively associated with an increasing risk of LD for all time lags. The highest RR and lowest AIC (Table 2) were observed with a delayed effect of 3 weeks (RR 1·05 for every 2 °C increase, 95% CI 1·03–1·07). The flexible curve representing the overall effect of weekly mean temperature with a 3-week lag on RR for all regions showed that a linear effect relationship existed between these two variables between, approximately, 5 °C and 17 °C (Fig. 2). Below 5 °C the curve was flat and the effect seemed to plateau and even decrease >20 °C. Flexible curves representing the overall effect of a standardized mean temperature on RR by month showed different patterns across the year (Fig. 4). In January, increasing mean temperature seemed to be associated with decreasing RR. From February to May and in September the effect was null. For the remaining months increasing mean temperature was associated with increasing RR. Unusually high weekly mean temperature (⩾20 °C) was statistically significantly negatively associated with an increasing risk of LD, with delayed effect of 1 week (RR 0·74, 95% CI 0·67–0·82).

### Atmospheric pressure

Weekly mean atmospheric pressure was negatively associated with an increasing risk of LD with delayed effect of 1 and 2 weeks. Both lowest RR and AIC (Table 2) were observed with a lagged effect of 1 week (RR 0·89 for every 5-hPa increase, 95% CI 0·87–0·91). The flexible curve showing the effect of mean atmospheric pressure with a lagged effect of 1 week on the RR suggested a linear relationship >1010 hPa (Fig. 1).

### Model and prediction

In the adjusted model meteorological variables were kept with the lag associated with the highest effect, as supported by calculated AIC. Calculated VIF did not indicate multicollinearity between these variables with values <10 (VIF 1·19 for weekly cumulative rainfall, 1·01 for weekly mean temperature, and 1·20 weekly mean atmospheric pressure). Thus, weekly cumulative rainfall with 1 week lag, weekly mean temperature with 3 weeks lag, and mean atmospheric pressure with 1 week lag were categorized and included in a model adjusted for region and seasonality (Table 3). Weekly cumulative rainfall was positively associated with an increasing notification rate of LD. With no weekly rainfall as a reference, the estimated RR of LD for weekly cumulative rainfall >40 mm with a lagged effect of 1 week was 2·14 (95% CI 1·90–2·42; rate 182 *vs*. 62 LD cases/10 million population). Weekly mean temperature was positively associated with an increasing notification rate of LD but RR did not increase further >20 °C. With weekly mean temperature <10 °C as a reference, the estimated RR of LD for weekly mean temperature of 15–19 °C with a lagged effect of 3 weeks was 2·00 (95% CI 1·75–2·28, rate 120 *vs*. 54/10 million population). Weekly mean atmospheric pressure was negatively associated with an increasing notification rate of LD. With weekly mean atmospheric pressure <1010 hPa as a reference, the estimated RR of LD for weekly mean atmospheric pressure of 1010–1019 hPa with a lagged effect of 1 week was 0·92 (95% CI 0·85–0·99, rate 86 *vs*. 88/10 million population).

**Table 3.** Estimated adjusted relative risk (RR) and 95% confidence interval (CI) of community-acquired Legionnaires' disease and potential confounders, number of cases and exposed, and rates per 10 million population, Denmark, Germany, Italy and The Netherlands, 2007–2012

	Exposed (million person-years)	No. of LD cases	Rate (cases/10 million pop.)	RR*	(95% CI)
Year					
2007	149·4	1042	70	1 (ref.)	
2008	158·5	1397	88	1·21	(1·11–1·32)
2009	158·5	1342	85	1·20	(1·10–1·32)
2010	158·5	1650	104	1·43	(1·31–1·56)
2011	158·1	1221	77	1·10	(1·00–1·20)
2012	157·5	1309	83	1·33	(1·21–1·45)
Weekly cumulative rainfall (1 week lag)					
0 mm	84·2	520	62	1 (ref.)	
1–9 mm	367·3	2507	68	1·16	(1·04–1·29)
10–19 mm	211·5	1493	71	1·31	(1·17–1·47)
20–29 mm	132·5	1274	96	1·69	(1·50–1·90)
30–39 mm	71·2	842	118	1·78	(1·57–2·02)
⩾40 mm	72·7	1325	182	2·14	(1·90–2·42)
Weekly mean temperature (3 weeks lag)					
<10 °C	419·2	2272	54	1 (ref.)	
10–14 °C	207·7	1611	78	1·38	(1·25–1·53)
15–19 °C	215·4	2582	120	2·00	(1·75–2·28)
20–24 °C	81·3	1310	161	1·74	(1·48–2·06)
⩾25 °C	16·4	186	113	1·78	(1·41–2·25)
Weekly mean atmospheric pressure (1 week lag)					
<1010 hPa	151·0	1336	88	1 (ref.)	
1010–1014 hPa	296·2	3081	104	1·00	(0·93–1·08)
1015–1019 hPa	286·5	2454	86	0·92	(0·85–0·99)
⩾1020 hPa	207·7	1090	52	0·93	(0·84–1·03)

LD, Legionnaires' disease; NUTS2, Nomenclature of Territorial Units for Statistics level 2; hPa, hectopascal.

*RRs from Poisson regression including covariates year (2007–2012), NUTS2 (one intercept for each region), population (offset parameter), weekly cumulative rainfall (1 week lag), weekly mean temperature (3 weeks lag), weekly mean atmospheric pressure (1 week lag), and adjusted for season using a cubic spline function with five knots.

### Interactions

There were positive interactions between increasing weekly mean temperature (3 weeks lag) and increasing weekly cumulative rainfall (1 week lag). The highest RR was found for weekly mean temperature of 15–19 °C and cumulative rainfall >30 mm compared to temperature <10 °C and rainfall <10 mm (RR 3·50, 95% CI 3·00–4·08, rate 189 *vs*. 47/10 million population). There were positive interactions between weekly cumulative rainfall (1 week lag) and mean atmospheric pressure (1 week lag) for rainfall ⩾20 mm and atmospheric pressure <1020 hPa. RRs ranged from 1·32 to 1·79 with overlapping 95% CIs (Supplementary material).

There were both negative and positive interactions between weekly mean temperature (3 weeks lag) and mean atmospheric pressure (1 week lag). Compared to weekly mean temperature <10 °C and atmospheric pressure <1010 hPa, temperature <10 °C and atmospheric pressure ⩾1020 hPa had a protective effect (RR 0·76, 95% CI 0·67–0·86, rate 48 *vs*. 64/10 million population). Increasing weekly mean temperature and decreasing mean atmospheric pressure were associated with higher risk of LD, peaking for temperature of 15–19 °C and atmospheric pressure <1010 hPa (RR 2·85, 95% CI 2·35–3·45, rate 135 *vs*. 64/10 million pop.) (Supplementary material).

### Sensitivity analyses

Overall, flexible curves representing the effect of meteorological variables on the RR showed similar shapes in all countries. However, the effect of weekly mean temperature (3 weeks lag) showed a higher RR in The Netherlands compared to the other three countries. The effect of weekly mean temperature seemed to plateau >20 °C in Italy but such mean temperatures were seldom observed in the other countries. When running the model for each country, estimated RRs by region were not significant in Denmark and The Netherlands. Conversely, estimated RRs varied across Italian and German regions. Neither the exclusion of Lombardy nor of Italy substantially changed the estimates of the final model.

## DISCUSSION

Using data from 77 regions across four countries we found an association between weekly cumulative rainfall, mean temperature, and atmospheric pressure and notification rate of community-acquired LD. The RR for LD after weekly cumulative rainfall >40 mm with a lagged effect of 1 week compared to no rainfall was 2·14 (95% CI 1·90–2·42). The RR for LD after weekly mean temperature of 15–19 °C with a lagged effect of 3 weeks compared to temperature <10 °C was 2·00 (95% CI 1·75–2·28). The effect of temperature plateaued and even decreased >20 °C. This was confirmed with the exploration of unusually high temperature in which the only statistically significant association observed was a protective effect of weekly mean temperature ⩾20 °C with a delayed effect of 1 week. The interaction between temperature and rainfall was also suggestive of a lesser effect of temperature >20 °C. This usually high temperature may well be associated with behavioural changes. In addition, our findings suggest that the effect of both temperature and rainfall may differ across the year with probably minimal effects during February–April. The protective effect of increased mean atmospheric pressure with a delay of 1 week was independent of the effect of rainfall.

The main strength of our study is the large sample size and the analysis of data collected in four different countries with the same data source. The relatively large number of LD cases included allowed for adjustment at the regional level and analysis by month. Since our analysis included countries covering a substantial part of Europe, both in terms of population and area, we think that our main findings could be extrapolated to other developed countries with comparable climates. By necessity, our analysis summarizes patterns over large regions. Our models allowed for different risks in different regions but the influences of weather exposures on LD notification rate were assumed equal. Our average effect may have diluted effects in smaller geographic regions. Furthermore, the geographical resolution was not perfect and we were probably not able to capture local weather conditions, especially in mountainous areas. However, these areas are unlikely to be densely populated and therefore to yield many LD cases. Last, some cases may have had a very brief incubation period and would therefore invalidate the 1-week lag assumption.

Our findings confirm the role of rainfall as an important environmental factor associated with an increased risk of community-acquired LD. This is in line with previous studies carried out in Spain and the United States [6, 25] and is supported by our understanding of the ecology of the bacteria [1]. The role played by temperature appear to be more complex with the highest risk found for a lag of 3 weeks. This sequence of warm weather followed by heavy rainfall as the most favourable conditions for community-acquired LD would support findings reported in The Netherlands [13]. The difficulty in determining the exact role of temperature may be due to the fact that mean weekly air temperature may imperfectly reflect daily and diurnal variations that impact *Legionella* growth. In addition, *Legionella* is not merely found in natural environments but also in man-made water systems where the temperature could be artificially modified. Unusually high temperature may be associated with long-lasting dryness not compensated for by rainfall in seasons with intense evaporation. The effect of the studied environmental factors on the protozoan hosts of *Legionella* may also play a role that should be investigated further [26, 27]. It may be that *Legionella* or its protozoan hosts have lower optimum temperature growth and range in some regions or under certain climates. There are also potential areas of confounding, especially for temperature and rainfall. High temperatures are more likely to be associated with different behaviours such as a higher frequency of outdoor activities or increased use of potentially hazardous environmental sources (e.g. showers or air conditioning). In addition, other potential sources, such as fountains or cooling towers, may be frequented more during warm weeks. Conversely, heavy rainfall could be associated with indoor activities but soon followed by increased outdoor activities. Unfortunately we were not able to investigate the role of humidity but since humidity is influenced by pressure and temperature we may have indirectly captured some of its potential effect. The understanding of the heterogeneity observed across countries and regions would benefit from further investigation. Depending on their geographical coordinates and terrain, countries may have different profiles in terms of temperature and rainfall. By adjusting for and analysing data by country we have tried to control for specificities unrelated to meteorological conditions, especially those related to surveillance systems. However, specific characteristics at both national and regional levels could explain some differences observed across countries. Thus, clinicians' awareness, use of diagnostic tests and reporting to the national public health authorities may differ across countries or even within a country. For example, a capture–recapture study carried out in Italy suggested higher underreporting in central-southern compared to northern regions [28].

Further studies should try to better understand the potential confounding effect of behavioural factors associated with community-acquired LD infections and their seasonal changes.

## CONCLUSION

Temperature, rainfall and atmospheric pressure are associated with LD risk. Community-acquired LD cases occurring during wintertime may be associated with sources less influenced by meteorological conditions. Unusually high temperatures were not associated with higher risk for LD.

## Supplementary material

For supplementary material accompanying this paper visit https://doi.org/10.1017/S0950268816001874. click here to view supplementary material
