# Supplementary material for: Short-term effects of atmospheric pressure, temperature, and rainfall on notification rate of community-acquired Legionnaires' disease in four European countries
Source: Epidemiol Infect. 2016 Aug 30;144(16):3483–93. doi: 10.1017/S0950268816001874 (PMC5111125; doi:10.1017/S0950268816001874)
Supplement: Supplementary file 1 [file S0950268816001874sup001.docx]

Supplementary Table S1. Estimated relative* risk and 95%CI of community-acquired Legionnaires’ disease for an interaction between weekly cumulative rainfall (one week lag) and weekly mean temperature (three weeks lag), Denmark, Germany, Italy and the Netherlands, 2007−2012

| Weekly cumulative rainfall (one week lag) | Weekly mean temperature (three weeks lag) | | | |
| --- | --- | --- | --- | --- |
|  | <10°C | 10 to 14°C | 15 to 19°C | ≥20°C |
| <10mm | 1 (ref.) | 1.20 (1.04-1.37) | 1.66 (1.42-1.95) | 1.50 (1.24-1.81) |
| 10 to 19mm | 1.13 (1.01-1.28) | 1.53 (1.31-1.79) | 2.00 (1.69-2.37) | 1.70 (1.38-2.11) |
| 20 to 29mm | 1.31 (1.15-1.50) | 1.82 (1.54-2.17) | 2.77 (2.34-3.27) | 2.66 (2.14-3.32) |
| ≥30mm | 1.37 (1.21-1.55) | 2.28 (2.00-2.61) | 3.50 (3.00-4.08) | 2.90 (2.38-3.54) |

Supplementary Table S2. Estimated relative risk* and 95%CI of community-acquired Legionnaires’ disease for an interaction between weekly cumulative rainfall (one week lag) and weekly mean atmospheric pressure (one week lag), Denmark, Germany, Italy and the Netherlands, 2007−2012

| Weekly cumulative rainfall (one week lag) | Weekly mean atmospheric pressure (one week lag) | | | |
| --- | --- | --- | --- | --- |
|  | <1010hPa | 1010 to 1014hPa | 1015 to 1019hPa | ≥1020hPa |
| <10mm | 1 (ref.) | 0.81 (0.66-0.99) | 0.91 (0.75-1.11) | 0.87 (0.71-1.06) |
| 10 to 19mm | 1.08 (0.85-1.37) | 1.09 (0.89-1.34) | 0.96 (0.78-1.19) | 1.08 (0.84-1.39) |
| 20 to 29mm | 1.32 (1.05-1.66) | 1.48 (1.20-1.82) | 1.37 (1.10-1.70) | 1.02 (0.74-1.42) |
| ≥30mm | 1.69 (1.38-2.07) | 1.79 (1.47-2.19) | 1.33 (1.07-1.65) | 1.34 (0.97-1.85) |

Supplementary Table S3. Estimated relative risk* and 95%CI of community-acquired Legionnaires’ disease for an interaction between weekly mean temperature (three weeks lag) and weekly mean atmospheric pressure (one week lag), Denmark, Germany, Italy and the Netherlands, 2007−2012

| Weekly mean temperature (three weeks lag) | Weekly mean atmospheric pressure (one week lag) | | | |
| --- | --- | --- | --- | --- |
|  | <1010hPa | 1010 to 1014hPa | 1015 to 1019hPa | ≥1020hPa |
| <10°C | 1 (ref.) | 0.89 (0.78-1.01) | 0.80 (0.71-0.92) | 0.76 (0.67-0.86) |
| 10 to 14°C | 1.60 (1.36-1.88) | 1.41 (1.21-1.64) | 1.23 (1.06-1.42) | 0.98 (0.81-1.18) |
| 15 to 19°C | 2.85 (2.35-3.45) | 2.22 (1.88-2.62) | 1.62 (1.37-1.91) | 1.34 (1.08-1.67) |
| ≥20°C | 2.03 (1.54-2.68) | 1.63 (1.34-1.99) | 1.52 (1.24-1.86) | 1.00 (0.64-1.56) |

*Relative risks from Poisson regression including covariates year (2007-2012), NUTS2 (one intercept for each region), population, weekly cumulative rainfall (one week lag), weekly mean temperature (three weeks lag), weekly mean atmospheric pressure (one week lag), adjusted for season using a cubic spline function with five knots, and an interaction term as indicated in the caption.
